# Supplementary material for: Climate windows of opportunity for plant expansion during the Phanerozoic
Source: Nat Commun. 2022 Aug 4;13:4530. doi: 10.1038/s41467-022-32077-7 (PMC9352767; doi:10.1038/s41467-022-32077-7)
Supplement: Supplementary file 1 — Supplementary Information [file 41467_2022_32077_MOESM1_ESM.pdf]

Supplementary Information

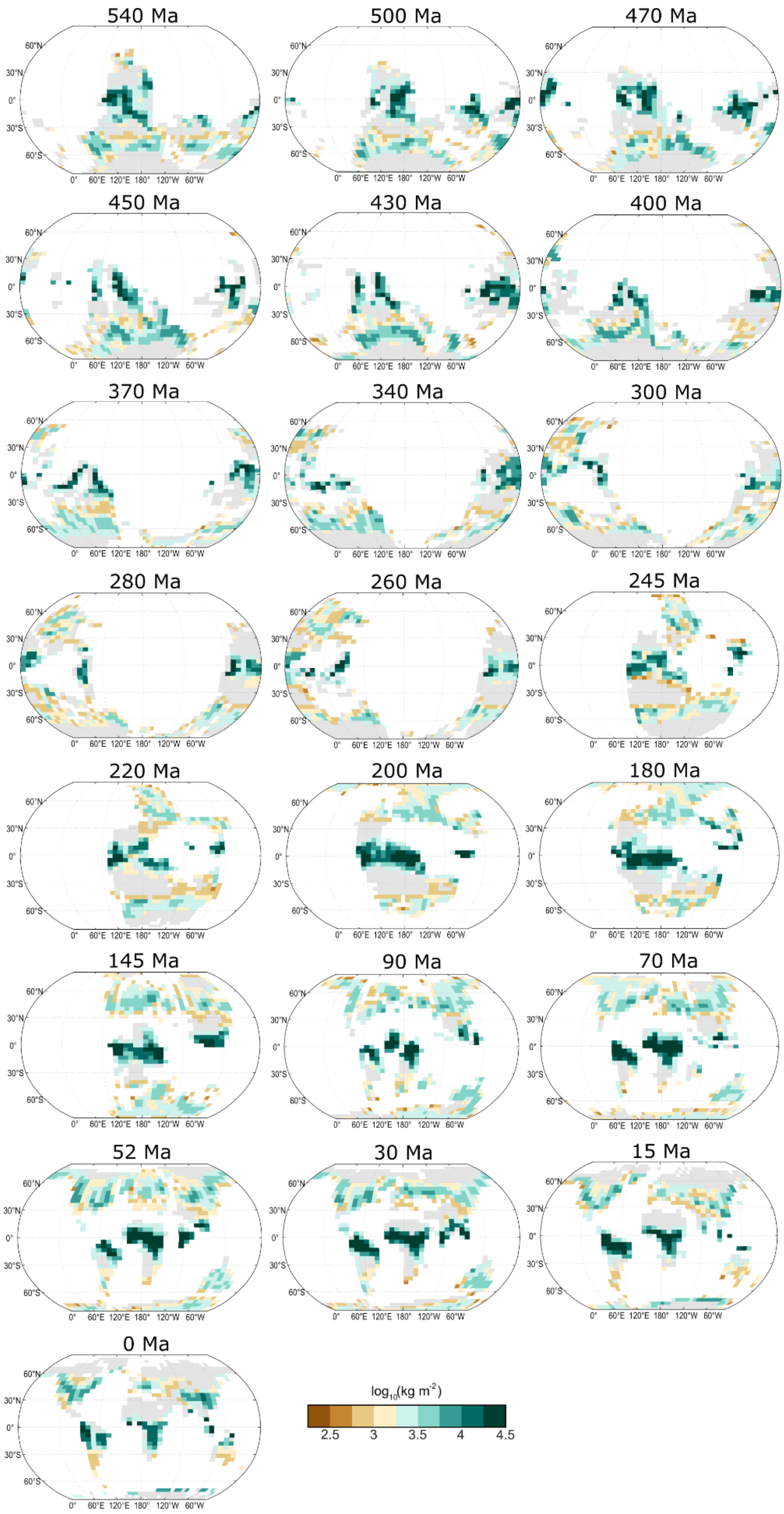

Figure S1. Potential biomass maps throughout the Phanerozoic. Maps showing terrestrial land (shaded grey) and potential biomass between 540-0 Ma (million years ago). The breakup of Pangea occurs between 200-180 Ma.

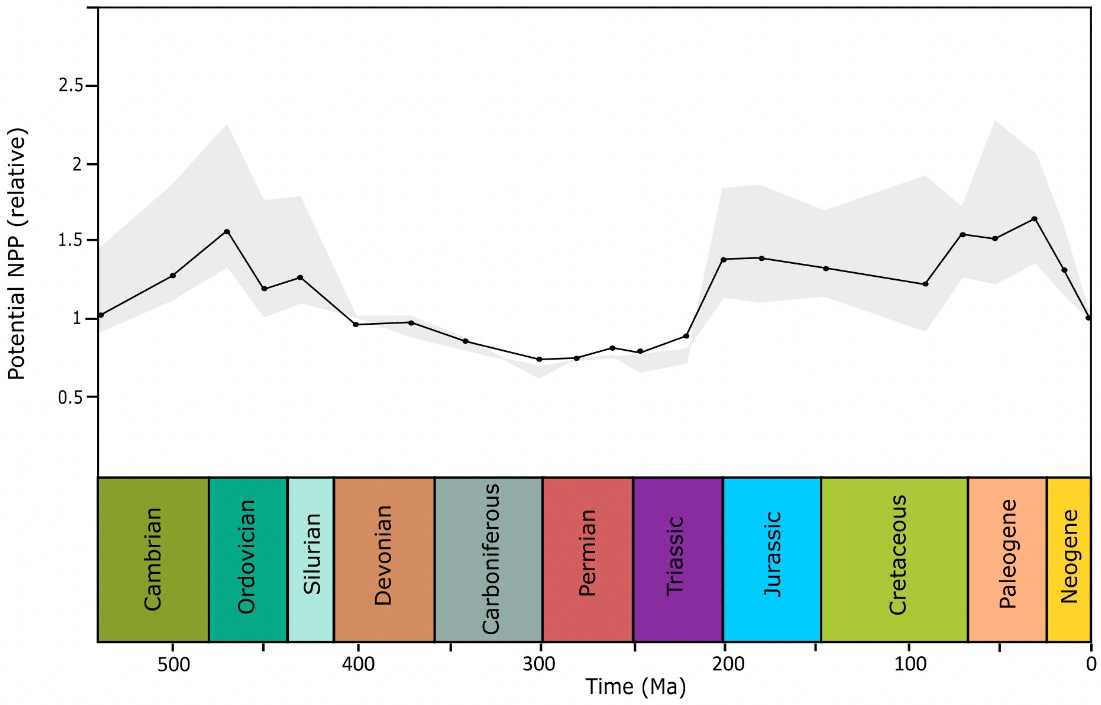

Figure S2. Potential NPP over the Phanerozoic. Relative to present day potential NPP. The pattern of NPP overtime matches that of biomass overtime due to the simplistic relationship between the two; a large portion of NPP is converted into biomass. Grey shaded area represents the min/max NPP obtained with the min/max CO_2 level used at each timepoint. Ma: million years ago.

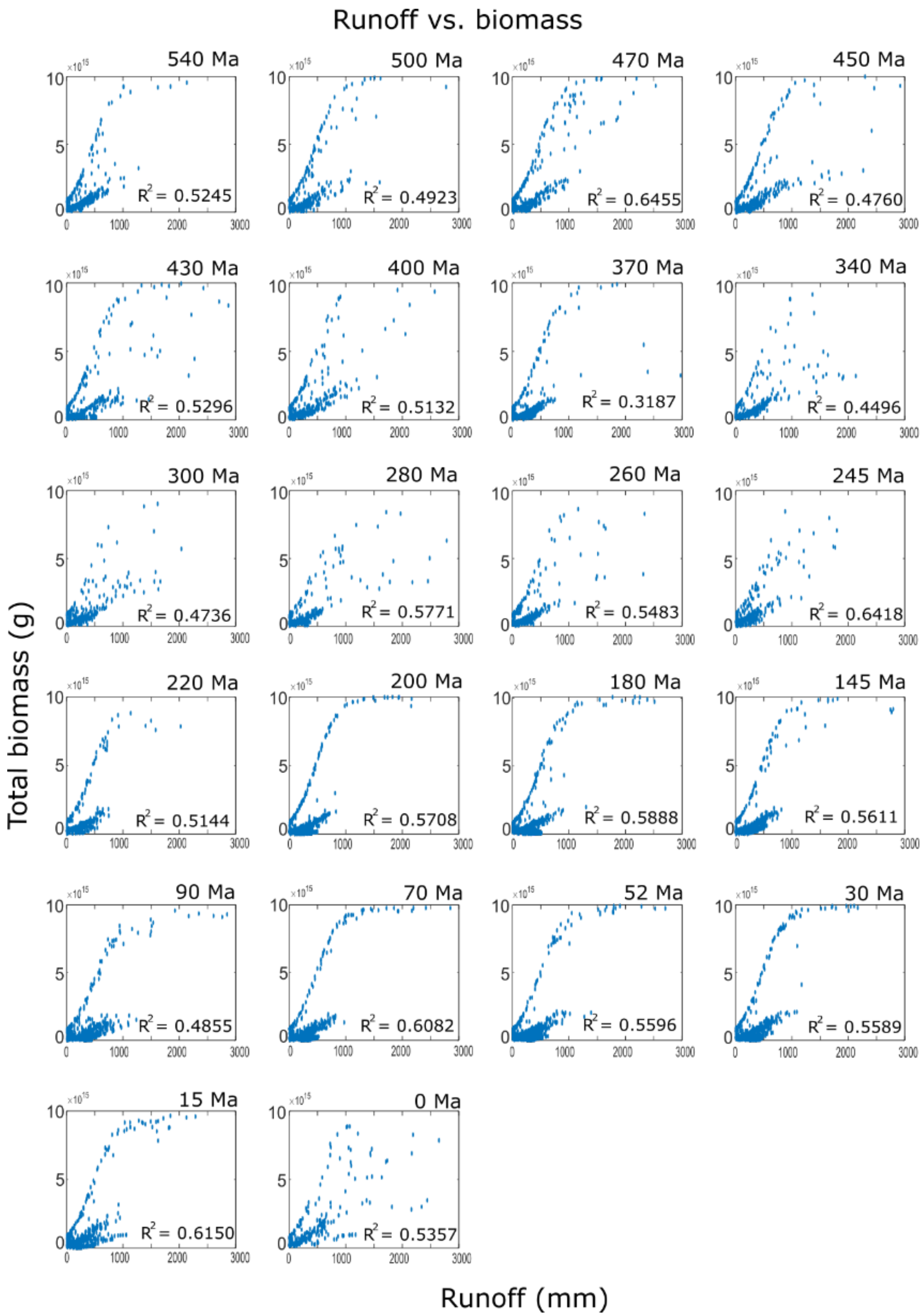
Runoff vs. biomass
540 Ma
R^2 = 0.5245
500 Ma
R^2 = 0.4923
470 Ma
R^2 = 0.6455
450 Ma
R^2 = 0.4760
430 Ma
R^2 = 0.5296
400 Ma
R^2 = 0.5132
370 Ma
R^2 = 0.3187
340 Ma
R^2 = 0.4496
300 Ma
R^2 = 0.4736
280 Ma
R^2 = 0.5771
260 Ma
R^2 = 0.5483
245 Ma
R^2 = 0.6418
220 Ma
R^2 = 0.5144
200 Ma
R^2 = 0.5708
180 Ma
R^2 = 0.5888
145 Ma
R^2 = 0.5611
90 Ma
R^2 = 0.4855
70 Ma
R^2 = 0.6082
52 Ma
R^2 = 0.5596
30 Ma
R^2 = 0.5589
15 Ma
R^2 = 0.6150
0 Ma
R^2 = 0.5357
Total biomass (g)
Runoff (mm)

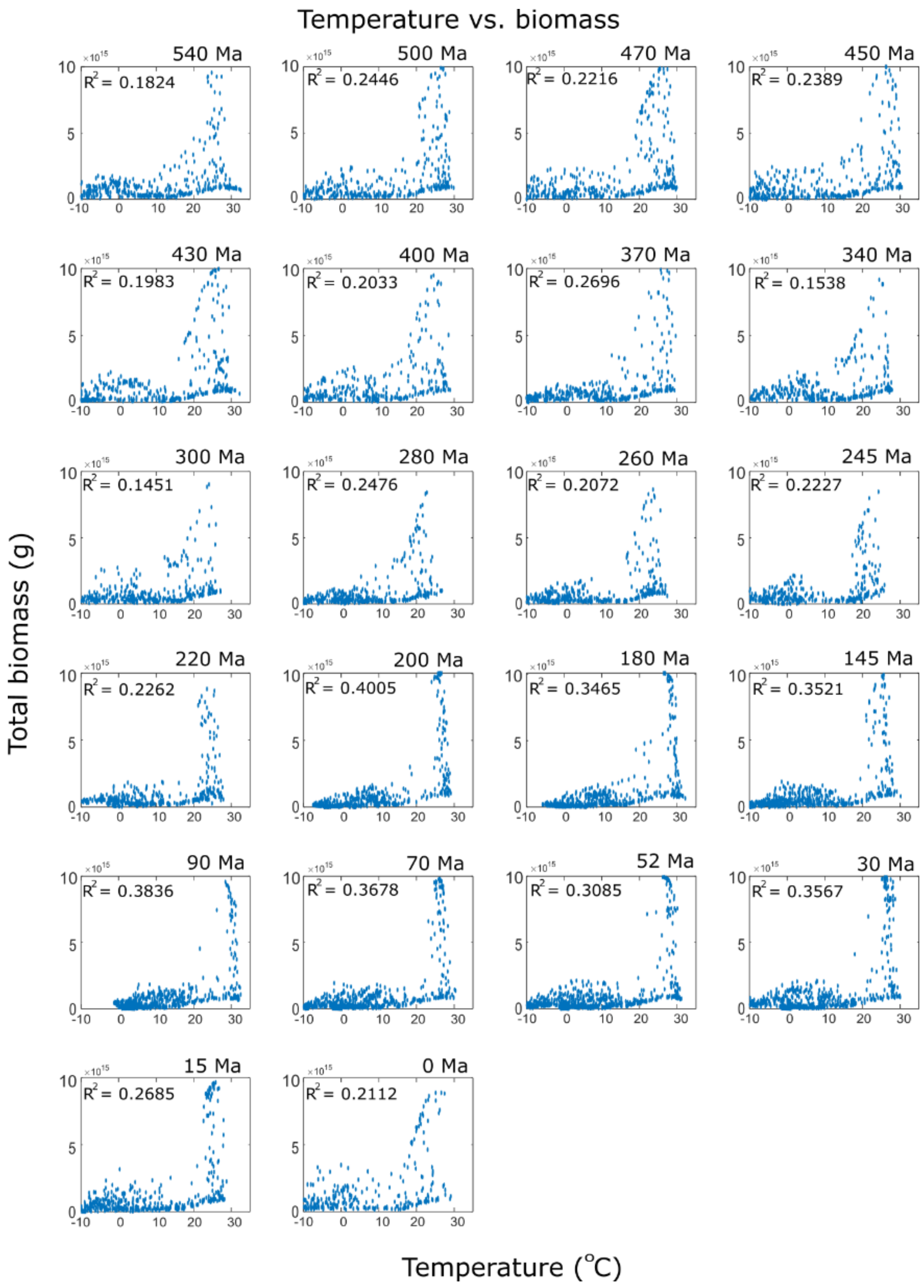

Figure S3. R-squared values between biomass and runoff, and temperature for each time point.

Average r-squared values are 0.5358 and 0.2615 respectively.

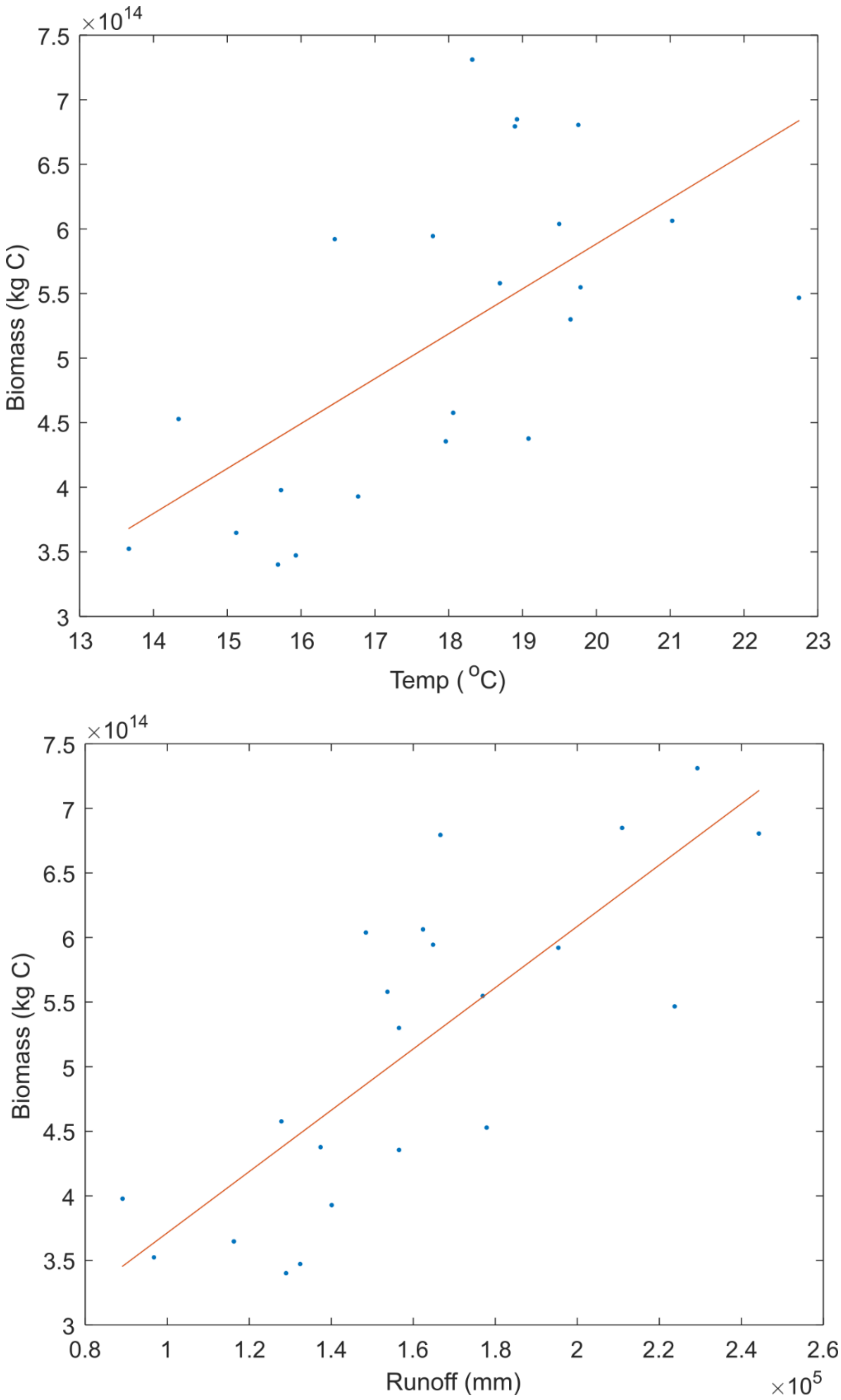

Figure S4. Linear regression between total global biomass, global temperature and global runoff. Using the 22 timepoints within the Phanerozoic, r-squared values are 0.399 and 0.623 for average global temperature and total global runoff, respectively.

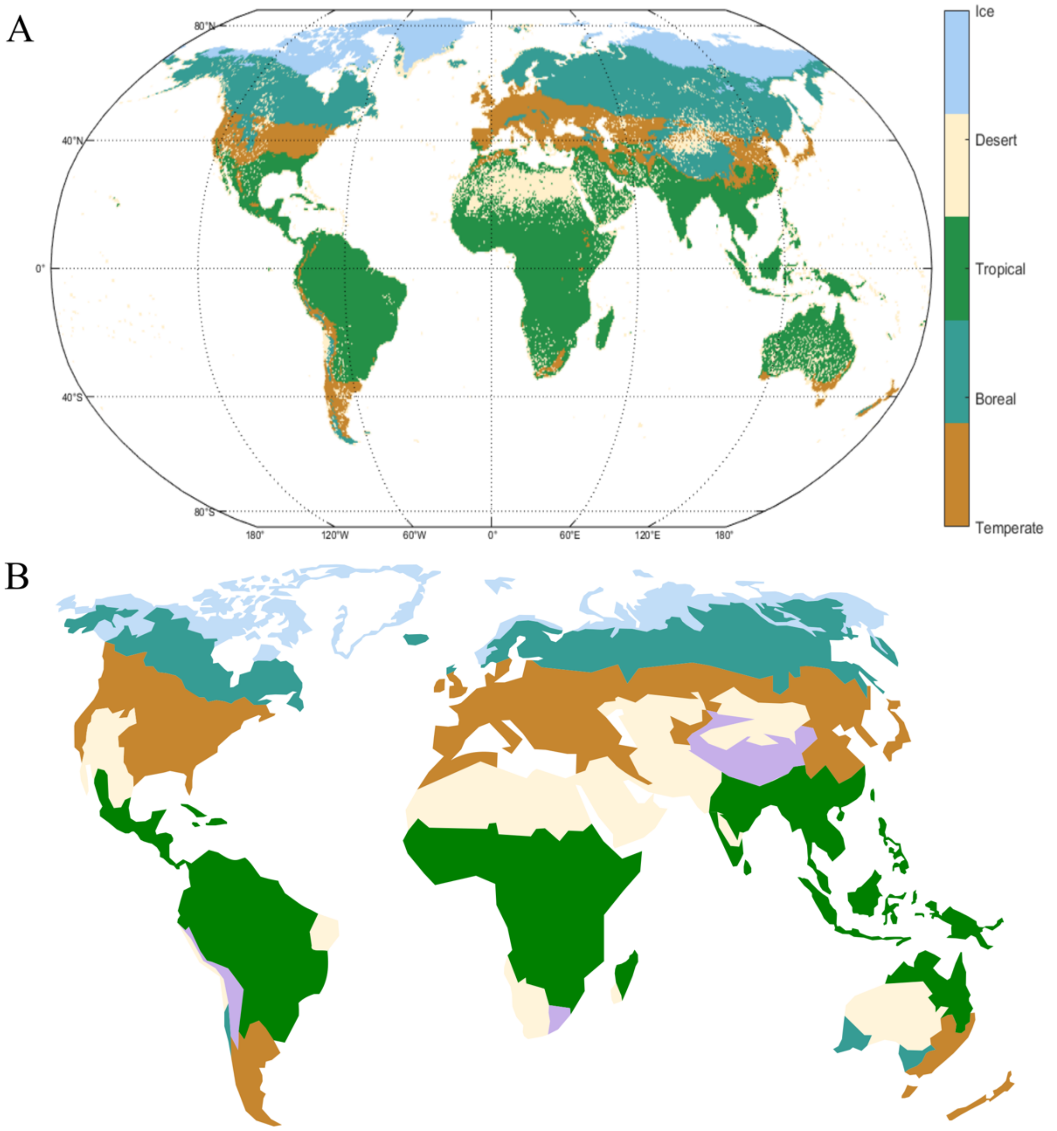

Figure S5. Comparison of biome distribution. A similar pattern is broadly observed between present day simplified biomes and FLORA predicted biomes. **(A)** Distribution of the three plant functional types (boreal, tropical and temperate) as predicted by FLORA using simple competition. Other biomes include the ice biome (land with temperatures < -10 °C) and desert biome (land where runoff <0) for present day. **(B)** Simplified map of present day terrestrial biomes modified from Trimble and Van Aarde (1). The original 14 biomes were condensed down to the five biomes used in FLORA, with the exception of the montane biome (purple).

Table S1. Associated parameters for plant functional types. Optimum temperature and temperature range (s.d: standard deviation), turnover ratio (f_{leaf}) and respiration rate (r) for each plant functional type.

**Plant functional type**	**Temperature optimum (ºC ± s.d)**	**Turnover ratio**	**Respiration rate (g C/g N/d)**
Tropical	27 ± 7	1	0.011
Temperate	15 ± 20	0.75	0.055
Boreal	0 ± 20	0.75	0.066

Table S2. FLORA biomass outputs for each time-point with CO_2 and O_2 levels. O_2 (kPa) (2), CO_2 level (ppm) used (3) and the sum of potential global biomass (Gt C) for each time point predicted by FLORA. Ma: million years ago.

**Time (Ma)**	**CO_2 (ppm)**	**O_2 (kPa)**	**Biomass (Gt C)**
540	5600	1.9	458
500	3360	5.14	558
470	3360	3.74	679
450	2800	5.05	530
430	2240	14.94	555
400	1680	16.76	436
370	1400	16.22	438
340	700	24.76	393
300	560	25.52	347
280	700	27.25	340
260	700	31.05	365
245	1120	30.77	352
220	1680	32.38	398
200	1680	28.6	604
180	1680	25.13	606
145	840	25.86	594
90	1120	26.65	547
70	560	27.69	685
52	700	25.12	681
30	560	21.41	731
15	360	21.57	592
0	280	20.72	453

References

1. Trimble, M. J. & Aarde, R. J. van. Geographical and taxonomic biases in research on biodiversity in human-modified landscapes. *Ecosphere* **3**, 1–16 (2012).

2. Krause, A. J. *et al.* Stepwise oxygenation of the Paleozoic atmosphere. *Nat. Commun. 2018 91* **9**, 1–10 (2018).

3. Mills, B. J. W. *et al.* Modelling the long-term carbon cycle, atmospheric CO2, and Earth surface temperature from late Neoproterozoic to present day. *Gondwana Res.* **67**, 172–186 (2019).
