# Supplementary material for: Climate windows of opportunity for plant expansion during the Phanerozoic
Source: Nat Commun. 2022 Aug 4;13:4530. doi: 10.1038/s41467-022-32077-7 (PMC9352767; doi:10.1038/s41467-022-32077-7)
Supplement: Supplementary file 3 — Description of Additional Supplementary Files [file 41467_2022_32077_MOESM3_ESM.pdf]

Description of Additional Supplementary Files

File Name: Supplementary Code 1
Description: Contains the MatLab code for FLORA, its validation and its run over the Phanerozoic. Parameter datasets are also included.
